# Delayed Development of a Giant Cranial Pseudomeningocele After Surgical Excision of a Parasagittal Meningioma: A Case Report

**DOI:** 10.7759/cureus.75372

**Published:** 2024-12-09

**Authors:** Kautilya R Patel, Himanshu YN, Alok M Uppar

**Affiliations:** 1 Department of Neurosurgery, National Institute of Mental Health and Neurosciences, Bengaluru, IND

**Keywords:** giant, meningioma, postoperative, pseudomeningocele, surgery

## Abstract

Pseudomeningoceles are among the most common postoperative neurosurgical complications, usually presenting in the early postoperative period and often responding well to nonsurgical management. Here, we present a case of a giant cranial pseudomeningocele that developed three years after parasagittal meningioma resection, without any known risk factors. Despite conservative measures, the pseudomeningocele grew significantly over two years, reaching 22 cm along its long axis. Immediate surgical management was necessary to address the underlying cause of the pseudomeningocele, alleviate the patient's discomfort, and restore her cosmesis. The pseudomeningocele, along with the redundant skin, was excised, followed by dural defect repair and cosmetic skin closure. The patient recovered well postoperatively. This case highlights the need for close follow-up of patients undergoing medical management for pseudomeningocele and timely adoption of surgical management when necessary.

## Introduction

Pseudomeningoceles are collections of cerebrospinal fluid (CSF) that leak through a dural defect into the subcutaneous tissue. They are among the most common postoperative complications following cranial neurosurgical procedures. The exact incidence of pseudomeningoceles after cranial surgeries is not well established, but studies report rates ranging from 5% to 13% across various types of surgeries [1,2]. They are more commonly seen following posterior fossa surgeries than supratentorial procedures. Most pseudomeningoceles respond well to nonsurgical treatments and rarely require surgical intervention. Spinal pseudomeningoceles larger than 8 cm are classified as giant pseudomeningoceles [3], but similar cranial pseudomeningoceles are extremely rare. Here, we present a unique case of a cranial pseudomeningocele that developed three years after surgery and progressively enlarged over the next two years. 

## Case presentation

A woman in her 30s was diagnosed with a left middle one-third parasagittal meningioma following evaluation for new-onset right focal motor seizures and right hemiparesis. She underwent a left middle one-third parasagittal craniotomy and Simpson’s grade 2 excision of the meningioma. The dura was primarily closed after the excision, and the bone flap was replaced and secured with silk threads. Her initial postoperative course was uneventful, with healthy wound healing. The histopathology report confirmed grade 1 fibroblastic meningioma. Consequently, she did not undergo adjuvant radiotherapy or chemotherapy and continued with serial MRI scans to monitor for recurrence. Three years after surgery, she noticed swelling at the surgical site and consulted the primary surgeon. The subgaleal collection was aspirated, and compression bandaging was recommended. The swelling progressively enlarged over the next two years, and she presented to our hospital five years after the index surgery. 

**Figure 1:**
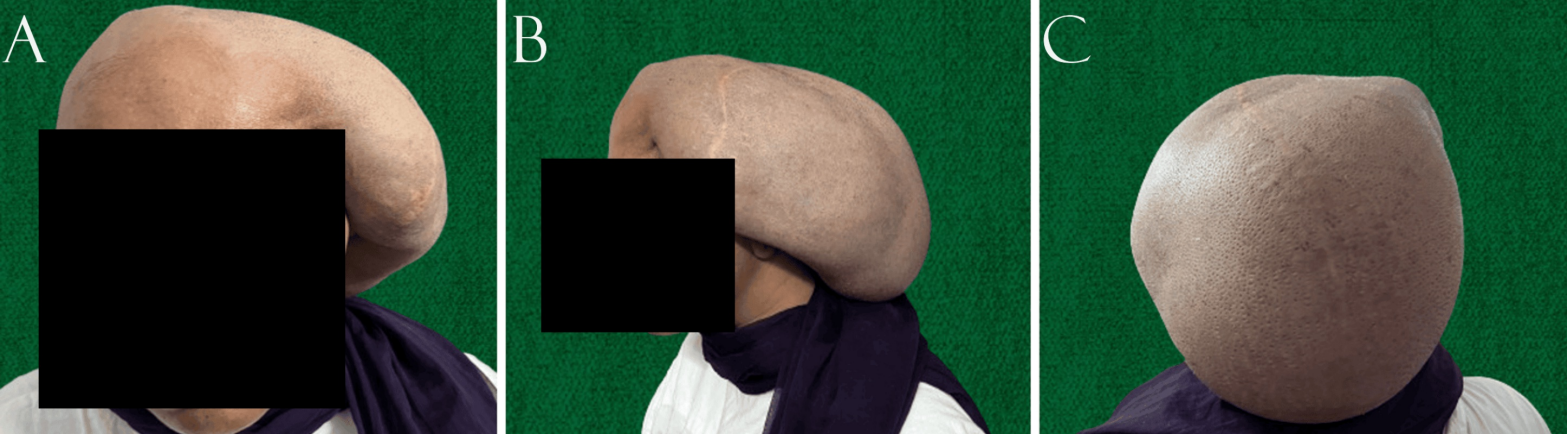
Clinical photographs of the patient showing the giant pseudomeningocele arising from the left side of the scalp (A) Anterior view. (B) Lateral view.  (C) Posterior view. Stretched-out surgical scars can be noted in panels (B) and (C). The swelling reaches up to the nape of the neck, as shown in panels (A) and (B).

**Figure 2:**
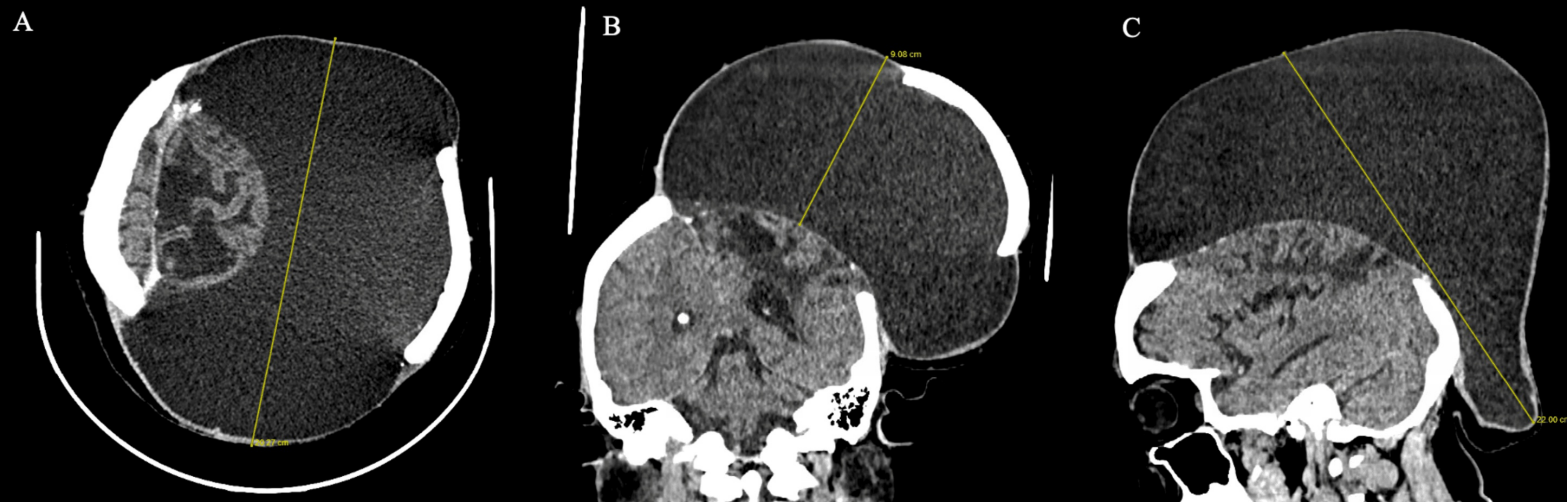
Head CT showing a large well-defined hypodense swelling arising from the craniotomy site (A) Axial section. (B) Coronal section. (C) Sagittal section. The swelling measures approximately 22 x 20 x 9 cm and is elevating the bone flap. Surgical site gliosis is noted.

At presentation, the patient complained of severe dragging pain due to the weight of the swelling. She had persistent right hemiparesis, with muscle strength of 4+/5 (Medical Research Council Scale) in both the right upper and lower limbs. Examination revealed a massive scalp swelling originating from the surgical scar, with intact overlying skin, as shown in Figure 1. The swelling measured 22 cm along its longest axis and extended to the nape of the neck. It was fluctuant and transilluminant and showed no signs of CSF leak. The craniotomy bone flap was palpable at the most convex part of the swelling. CT imaging (Figure 2) revealed a large hypodense fluid collection arising from the craniotomy site, with the bone flap adherent to the wall of the swelling. There was no evidence of hydrocephalus, and the underlying brain parenchyma showed gliosis without any enhancing lesion.

The patient underwent re-exploration of the previous surgical scar with excision of the pseudomeningocele. A lumbar drain was placed preoperatively. She was positioned on a horseshoe headrest with the pseudomeningocele lying dependent on the left side. After opening the surgical scar, about 1.2 L of xanthochromic fluid was drained. The bone flap was separated from the wall of the swelling, and the redundant skin was excised. A dural defect in the parasagittal region was reinforced with a galeal graft harvested from the excised skin and fibrin glue. The bone flap was secured with rigid titanium fixation plates and screws. The scalp defect was primarily closed after excision of the redundant skin. Vitals remained stable intraoperatively, and the lumbar drain was removed on postoperative day two. The surgical wound healed well, and the patient recovered uneventfully. There was no recurrence of the pseudomeningocele or CSF leak at the three-month follow-up.

## Discussion

This case report describes the delayed development of a massive pseudomeningocele in a young patient with no known risk factors. Giant postoperative cranial pseudomeningoceles are extremely rare entities, and to the best of our knowledge, this is the largest cranial pseudomeningocele reported in the literature. Patients usually present with pseudomeningoceles within a year of the index surgery. Known risk factors for the development of postoperative pseudomeningoceles include poor wound strength due to adjuvant radiotherapy, malnutrition, local infection, posterior fossa surgery, increased CSF pressure from hydrocephalus, and communication between the operative cavity and the ventricles. However, pseudomeningoceles are generally self-limited and resolve spontaneously or with nonsurgical interventions [4].

Our patient developed swelling at the operative site three years after the index surgery, which is unusually late. Attempts at conservative management with aspiration and bandaging were unsuccessful, and the pseudomeningocele progressively enlarged despite the absence of known risk factors. A possible explanation for this is poor healing of the durotomy margins, leading to the development of rent with a one-way valve mechanism that allowed progressive CSF accumulations. High CSF pressure beneath the bone flap likely caused the failure of thread fixation, preventing bone fusion and lifting the bone flap into the wall of the pseudomeningocele.

There is no standard treatment protocol for the management of pseudomeningocele. In the absence of hydrocephalus, most clinicians follow conservative or medical management, expecting spontaneous resolution in most cases. Management strategies include percutaneous aspiration and compression bandaging, acetazolamide with compression bandage, continuous or intermittent lumbar drainage, and subgaleal-peritoneal shunt [5]. However, the maximum duration of conservative management before transitioning to surgical management is not clearly defined. Tran et al. reported spontaneous resolution of pseudomeningocele 15 months after the index surgery [4]. In cases with hydrocephalus, CSF diversion via external ventricular drainage, ventriculoperitoneal shunt, or third ventriculostomy may be attempted. Failure of these measures requires re-exploration and repair of the dural defect. We adopted an aggressive surgical approach due to the pseudomeningocele's unusual size and the patient's significant discomfort, including dragging pain and cosmetic disfigurement. This strategy allowed us to address the underlying cause of the pseudomeningocele and restore the patient’s cosmesis through fluid drainage and excision of the redundant scalp skin.

This case highlights the rare possibility of delayed development of pseudomeningocele after cranial surgery in the absence of known risk factors and the possibility of undue progression without aggressive management. It reinforces the need for close follow-up of patients undergoing medical management for pseudomeningocele and timely surgical intervention to prevent further progression.

## Conclusions

Cranial pseudomeningoceles can have a delayed development after the index surgery and may enlarge to unusual dimensions in the absence of known risk factors. Pseudomeningoceles that enlarge and do not respond to initial medical management should be considered for timely surgical intervention.
